# Effect of inverse ratio ventilation on hemodynamics and respiratory mechanics in obese patients undergoing laparoscopic sleeve gastrectomy

**DOI:** 10.1186/s42077-021-00152-8

**Published:** 2021-04-13

**Authors:** Noha Hussein Sayed, Mohamed Saeed Abd Elaziz, Amgad Serag Elkholy, Mohamed Osman Taeimah

**Affiliations:** Department of Anesthesiology, Intensive Care and Pain Management, Faculty of Medicine, Ain-Shams University, Abbassia, Cairo, 11591 Egypt

**Keywords:** Inverse ratio ventilation, Morbid obesity, Laparoscopic sleeve gastrectomy, Respiratory mechanics

## Abstract

**Background:**

Obesity has become a global epidemic problem affecting every system and is associated with many consequences including coronary artery disease, hypertension, diabetes mellitus, dyslipidemia, obstructive sleep apnea, and socioeconomic and psychosocial impairment. Laparoscopic sleeve gastrectomy is one of the best and most commonly done operations for weight loss. Elevated peak airway pressure and hypoxemia are common problems that anesthesiologists face during laparoscopic surgeries with conventional volume-controlled ventilation. This study aimed at the use of the prolonged I:E ratio as an alternative strategy to improve gas exchange and the respiratory mechanics of obese patients undergoing laparoscopic sleeve gastrectomy.

**Results:**

The study was a prospective randomized controlled trial and was performed between April 2019 and March 2020. After the approval of the departmental ethical committee and the informed written consent had been taken from the patients, fifty-two obese patients undergoing laparoscopic sleeve gastrectomy were enrolled in this study. After endotracheal intubation, the patients were randomly divided into the IRV group (*n*=26) and the VCV group (*n*=26). Respiratory parameters were adjusted as tidal volume (Vt) 8mL/kg ideal body weight, respiratory rate 12 breaths/min, positive-end expiratory pressure (PEEP) 0, fractional inspired oxygen (FiO_2_) 0.6, and I:E ratio 1:2 for the VCV group and 2:1 for the IRV group; hemodynamics and respiratory mechanics were monitored and recorded after intubation (0 min), before pneumoperitoneum (10 mins), and after pneumoperitoneum (20 mins), 30, 40, 50, and 60 mins. IRV significantly improves the respiratory mechanics during pneumoperitoneum in the form of decreasing the peak pressure (Ppeak) and plateau pressure (Pplat) and improving the dynamic compliance, but the mean pressure (Pmean) was increased; it also increased the partial pressure of oxygen (arterial PO_2_) significantly. No statistical significance was found regarding the demographic data or the hemodynamics.

**Conclusion:**

IRV is superior to conventional VCV in morbidly obese patients undergoing laparoscopic sleeve gastrectomy as it improves respiratory mechanics and oxygenation.

## Background

Obesity is an abnormal or excessive fat accumulation that may impair health. A simple measure for classifying obesity in adults is body mass index( BMI). The WHO categorizes obesity as grade I obesity (BMI 30–34 kg/m^2^), grade II obesity (BMI 35–39 kg/m^2^), or grade III obesity (BMI at least 40 kg/m^2^) (Ng et al. 2014).

Several factors result in obesity: genetic factor, levels of physical activity, diet, and cultural and social factors. Several co-morbidities are associated with obesity including cardiovascular disease, hypertension, diabetes, obstructive sleep apnea (OSA), dyslipidemia, gastroesophageal reflux disease (GERD), anxiety, and depression (WHO Fact Sheet, 2018).

When conservative measures of weight management fail, bariatric surgery becomes the most effective and safe treatment for morbid obesity, especially with established safety and significant reduction of co-morbidities such as diabetes and hypertension (Schiavon et al. 2018).

Pelosi et al. (1998) pointed out that obesity was directly related to respiratory airway resistance and inversely related to oxygenation, functional residual capacity, and pulmonary compliance. Eichenberger et al. (2002) also demonstrated that atelectasis following general anesthesia persisted for at least 24 h in morbidly obese patients, while it resolved in a shorter time in normal-weight patients postoperatively (Hu et al. 2016).

Sleeve gastrectomy is performed with the patient in the supine position, open legs, and in reverse Trendelenburg position with an angle of 30°. The assistant surgeon and the scrub nurse are on his right side; the main surgeon is positioned between the lower limbs (Ramos et al., 2015a, b).

Laparoscopic sleeve gastrectomy is done by the dissection of the pad of fat of the esophagogastric junction, to allow complete visualization of the left diaphragmatic crus. Then, release and ligation of the great gastric curvature with stablers starting at the distal portion of the gastric body, continuing into the esophagus, and then distal to the pylorus (Basha et al. 2014).

Laparoscopic surgery is known to have adverse effects on respiratory mechanics and gas exchange. The increased intra-abdominal pressure caused by insufflation of carbon dioxide (CO_2_) results in a cephalad shift of the diaphragm, which leads to a decrease in lung volume and atelectasis (Carron et al. 2010).

It might be difficult to improve the gas exchange simply by changing the tidal volume (VT) or respiratory rate, as the high airway pressure may not allow a further increase in VT, and increasing the respiratory rate sometimes fails to correct hypercapnea. Thus, CO_2_ retention that occurs with a high airway pressure may make the anesthetic management challenging especially for morbidly obese patients (Sinha et al. 2012).

Prolongation of inspiratory to expiratory ratio has become an alternative strategy for improving gas exchange and respiratory mechanics. This strategy is used in surgical patients with reduced lung compliance due to surgical factors during general anesthesia as well as critical patients with acute respiratory syndrome (Talab et al. 2009).

## Aim of the study

This study was designed to investigate whether volume-controlled inverse ratio ventilation (IRV) with inspiratory to expiratory (I:E) ratio of 2:1 could improve oxygenation as a primary outcome in morbidly obese patients undergoing laparoscopic sleeve surgery, and the effect of IRV on respiratory mechanics (peak pressure, plateau pressure, and compliance ) and hemodynamics as a secondary outcome

## Methods

The study was a prospective randomized controlled trial and was performed between April 2019 and March 2020. After the approval of the departmental ethical committee and the informed written consent had been taken from the patients, we chose a total of 52 morbidly obese patients for a laparoscopic sleeve gastrectomy in reverse Trendelenburg position, from 21 to 45 years old, ranging BMI between 35 and 50 kg/m^2^, ASA II and III. We excluded patients with age more than 45 years and less than 21 years, BMI higher than 50kg/m^2^, ASA class IV, severe intraoperative bleeding affecting hemodynamics, and operations that extended more than 60 min. The 52 patients were randomly divided into 2 groups: IRV (*n*=26) and VCV (*n*=26).

In the OR, all patients were premedicated with prokinetics intravenous metoclopramide 20mg and H_2_ blockers ranitidine 50 mg. Monitoring, including electrocardiogram, invasive blood pressure, pulse oximetry, and venous access, was established. After preoxygenation for 3–5 min, anesthesia was induced with intravenous fentanyl 1–2 ug/kg lean body weight (LBW), propofol 2 mg/kg LBW given over 15 s, and atracurium 0.50 mg/kg ideal body weight (IBW), followed by paracetamol 10mg/kg IBW. After tracheal intubation using a direct laryngoscope, capnography was established and the lungs were ventilated with a Datex-Ohmeda Aespire anesthesia ventilator. Ventilation parameters were set as tidal volume 8 mL/kg ideal body weight, respiratory rate 12 breaths/min, 0 PEEP, fraction inspired oxygen (FiO_2_) 0.6, oxygen flow 2 L/min, inspiratory pause 10%, and an I:E ratio of 2:1 (in the IRV group) or 1:2 (in the control group). Anesthesia was maintained with 1.5–2.5 vol% end-tidal isofluorane to keep control of the hemodynamic response (blood pressure and heart rate) within a 25% range of the preoperative value. Trocars were placed in a supine position. Pneumoperitoneum tension was set at 15 mmHg. Lactated Ringer’s solution was infused at a rate of 6–8 mL/kg/h throughout the study. The same team of surgeons conducted the operations and were unaware of the patient’s study group. After surgery, all patients were extubated at the operation room and transferred to the post-anesthesia care unit.

### Measurements

Lung mechanics readings including peak pressure (Ppeak), plateau pressure (Pplat), and mean airway pressure (Pmean) were recorded every 10 min till the end of the first hour; also, dynamic and static compliance of the respiratory system were calculated after intubation and every 10 min until the end of the first hour: static compliance = tidal volume/Pplat − PEEP and dynamic compliance = tidal volume/Ppeak − PEEP.

Hemodynamic parameters, mean arterial blood pressure (MAP), and heart rate (HR) were monitored at the start and all through the procedure and recorded before anesthesia induction (T0), immediately after intubation (T1), before pneumoperitoneum (T2), after pneumoperitoneum (T3), and then every 10 to 60 min.

Arterial blood gas was analyzed using a blood gas analyzer at T0 and *every 10 min* in the first hour for arterial oxygen tension (arterial PaO_2_).

### Sample size

#### Twenty-six patients in each group (total 52 patients)

In this clinical trial, we have used oxygenation (represented as PaO_2_) as a primary outcome to compare the effect of the two methods of ventilation. Using two independent samples *t* test with expected large effect size (0.8), a level of significance of ≤0.05, and a power of 0.80, a sample size of at least 26 cases per group is needed**.** The patients were randomized using a random number table and the use of a closed envelope technique which was employed to allocate patients between the two groups.

##### Statistical analysis

Data were analyzed using the Statistical Package for Social Science (SPSS) version 22.0. Quantitative data were expressed as mean ± standard deviation (SD). Qualitative data were expressed as frequency and percentage.

The following tests were used:

▪ The independent-samples *t* test of significance was used when comparing between two means.

▪ The chi-square (*χ*^2^) test of significance was used in order to compare proportions between two qualitative parameters.

▪ The confidence interval was set to 95%, and the margin of error accepted was set to 5%. So, the *P*-value was considered significant as follows:

▪ Probability (*P*-value)

- *P*-value <0.05 was considered significant.
- *P*-value <0.001 was considered as highly significant.
- *P*-value >0.05 was considered non-significant.

## Results

### Demographic data

Statistical analysis for demographic data for the two groups revealed that there was no statistically significant difference (*P*-value >0.05), and there was no statistically significant difference in the duration of surgery as shown in Table 1.

**Table 1:** Comparison between the two studied groups as regards demographic data

	VCV group (*N*=26)	IRV group (*N*=26)	*P*-value
Age (years)	34.42 **±** 6.836	33.58 **±** 6.113	0.640
BMI (kg/m^2^)	41.38 **±** 3.44	41.66 **±** 4.19	0.797
Sex (M/F)	15/11	12/14	0.5788
Duration of surgery (min)	63.54±4.5	65.32±3.8	0.1296

*P* is significant when *P*≤ 0.05

### Partial pressure of oxygen

Regarding PO_2_, on comparison of the two groups, there was no statistically significant differences at T0 (preoperatively) and T1 (just after intubation) (*P*-value >0.05), but starting from T2 (just before pneumoperitoneum) to T6 after (60 min), there was a statistically significant difference in PO_2_ between the two groups as seen in Table 2.

**Table 2:** Comparison between the two studied groups as regards partial pressure of oxygen (PO_2_)

PO_2_			
Partial pressure of oxygen (mmHg)	VCV group (*N*=26)	IRV group (*N*=26)	*P*-value
Preoperative (T0)	79.27 ± 3.91	80.69 ± 4.33	0.220
PO_2_ (after intubation) (T1)	131.13 ± 5.57	131.19 ± 5.27	0.939
PO_2_ before pneumoperitoneum (T2)	181.46 ±7.24	188.31 ± 5.72	0.0001*
PO_2_ JUST after pneumoperitoneum (T3)	179.58 ± 7.65	191.88 ± 5.47	0.0001*
PO_2_ (T4)	177.38 ± 7.66	189.00 ± 5.51	0.0001*
PO_2_ (T5)	178.65 ± 8.24	194.04 ± 7.06	0.0001*
PO_2_ (T6)	178.27 ± 8.45	194.62 ± 6.15	0.0001*

*P* is significant when *P*≤ 0.05

### Peak airway pressure

Regarding the peak airway pressure, our study showed that there was a statistically significant difference between the two groups with generally lower peak airway pressure at the study group (IRV) than the control group (VCV) as shown in Table 3.

**Table 3:** Comparison between the two studied groups as regards peak airway pressure

Peak airway pressure			
Peak pressure (cmH_2_O)	VCV group (*N*=26)	IRV group (*N*=26)	*P*-value
Peak 1 (after intubation)	20.31 ± 2.02	18.35 ± 2.04	0.001*
Peak 2 (before pneumoperitoneum)	20.00 ± 1.166	17.19 ± 1.327	0.0001*
Peak 3 (after pneumoperitoneum)	30.15 ± 1.85	27.12 ± 1.423	0.0001*
Peak 4	28.19 ± 1.88	24.54 ± 1.77	0.0001*
Peak 5	27.58 ± 1.55	24.08 ± 1.495	0.0001*
Peak 6	27.62 ± 1.96	24.58 ± 1.33	0.0001*

*P* is significant when *P*≤ 0.05

#### Plateau pressure

The results of the study including plateau pressure revealed a statistically significant difference between both groups through all times (*P*-value <0.05) with lower plateau pressure in the study group than in the control group as shown in Table 4.

**Table 4:** Comparison between the two studied groups as regards plateau pressure

Plateau airway pressure			
Plateau pressure (cmH_2_O)	VCV group (*N*=26)	IRV group (*N*=26)	*P*-value
Plateau 1 (after intubation)	17.69 ± 2.11	15.88 ± 2.18	0.004*
Plateau 2 (before pneumoperitoneum)	17.08 ± 1.29	14.77 ± 1.31	0.0001*
Plateau 3 (after pneumoperitoneum)	26.88 ± 1.84	23.73 ± 1.37	0.0001*
Plateau 4	25.00 ± 1.83	21.12 ± 2.12	0.0001*
Plateau 5	24.38 ±1.75	20.42 ± 1.42	0.0001*
Plateau 6	24.35 ± 2.06	20.77 ± 1.28	0.0001*

*P* is significant when *P*≤ 0.05

#### Mean airway pressure

As regards the mean airway pressure, this study revealed that there is a statistically significant difference between the two groups (*P*-value <0.05) with the study group higher than the control group as shown in Table 5.

**Table 5:** Comparison between the two studied groups according to the mean airway pressure

Mean airway pressure				
Mean airway pressure (cmH_2_O)	VCV group (*N*=26)	IRV group (*N*=26)	*T*	*P*-value
Pmean T1 (after intubation)	6.73 ± 0.827	9.38 ± 1.06	10.056	0.0001*
Pmean T2 (before pneumoperitoneum)	6.69 ± 0.47	8.85 ± 0.732	12.623	0.0001*
Pmean T3 (after pneumoperitoneum)	10.04 ± 0.72	13.77 ± 0.765	18.113	0.0001*
Pmean T4	9.35 ± 0.75	12.46 ± 0.95	13.18	0.0001*
Pmean T5	9.15 ± 0.54	12.27 ± 0.78	16.75	0.0001*
Pmean T6	9.12 ± 0.65	12.46 ± 0.65	18.57	0.0001*

### Dynamic compliance

In comparison with the results of this study as regards the dynamic compliance (CD), we observed a statistically significant difference (*P*-value < 0.05) between the two groups at all times as seen in Table 6.

**Table 6:** Comparison between the two studied groups as regards dynamic compliance

Dynamic compliance			
Dynamic compliance (mL/cmH_2_O)	VCV group (*N*=26)	IRV group (*N*=26)	*P*-value
CD (after intubation) T1	24.96 ± 3.33	27.38 ± 3.53	0.014*
CD (before pneumoperitoneum) T2	25.46 ± 4.75	29.31 ± 4.92	0.006*
CD ( after pneumoperitoneum) T3	16.58 ± 2.69	18.58 ± 3.51	0.025*
CD T4	18.23 ± 3.65	20.5 ± 4.03	0.038*
CD T5	18.62 ± 3.44	20.89 ± 4.1	0.036*
CD T6	18.58 ± 3.64	21.23 ± 3.34	0.009*

*P* is significant when *P*≤ 0.05

#### Static compliance

Regarding the static compliance, these study results revealed a statistically significant difference between the study and the control groups with the study group showing higher compliance as seen in Table 7.

**Table 7:** Comparison between the two studied groups as regards static compliance

Static compliance			
Static compliance (mL/cmH_2_O)	VCV group (*N*=26)	IRV group (*N*=26)	*P*-value
CS (after intubation) T1	28.62 ± 3.68	31.65 ± 4.75	0.013*
CS (before pneumoperitoneum) T2	29.81 ± 5.52	34.08 ± 5.9	0.009*
CS (after pneumoperitoneum) T3	19.08 ± 3.44	21.23 ±4.02	0.043*
CS T4	20.46 ± 4.18	24.08 ± 5.15	0.008*
CS T5	21.04 ± 3.93	24.69 ± 4.83	0.004*
CS T6	21. ± 4.3	24.35 ± 4.95	0.012*

*P* is significant when *P*≤ 0.05

### Vital data

#### Mean arterial pressure

Regarding mean arterial pressure, we observed that the difference was statistically non-significant at T0 or later on after intubation and after pneumoperitoneum as seen in Table 8.

**Table 8:** Comparison between the two studied groups as regards mean arterial pressure

MAP			
Mean arterial pressure (mmHg)	VCV group (*N*=26)	IRV group (*N*=26)	*P*-value
Preoperative T0	70.46 ± 5.54	68.37 ± 4.3	0.217
MAP (after intubation) T1	75.35 ± 5.2	73.65 ± 3.05	0.158
MAP (before pneumoperitoneum) T2	68.81 **±** 6.29	67.19 ± 5.65	0.334
MAP (after pneumoperitoneum) T3	72.04 ± 6.302	70.77 ± 6.07	0.463
MAP T4	69.19 ± 5.85	69.69 ± 5.18	0.746
MAP T5	67.04 ± 4.75	67.31 ± 4.22	0.830
MAP T6	78.77 ± 4.11	79.12 ± 7.81	0.842

*P* is significant when *P*≤ 0.05

#### Heart rate

When comparing heart rate changes between the two groups, we observed that there was no statistically significant difference as shown in Table 9.

**Table 9:** Comparison between the two studied groups as regards heart rate (HR)

Heart rate			
Heart rate (BPM)	VCV group (*N*=26)	IRV group (*N*=26)	*P*-value
Preoperative T0	76.35 ± 3.64	78.92 ± 5.75	0.059
HR (after intubation) T1	86.81 ± 5.08	89.65 ± 8.90	0.163
HR (before Pneumoperitoneum) T2	81.92 ± 5.93	82.73 ± 7.14	0.659
HR (after pneumoperitoneum) T3	78.54 ± 6.45	79.31 ± 7.9	0.702
HR T4	78.65 ± 6.07	77.96 ± 6.68	0.697
HR T5	79.92 ± 4.97	80.42 ± 7.83	0.785
HR T6	81.23 ± 4.62	81.27 ± 8.15	0.983

*P* is significant when *P*≤ 0.05

## Discussion

Obesity is one of the most major health problems affecting every system and is associated with many consequences including an increased incidence of coronary artery disease, hypertension, diabetes mellitus, dyslipidemia, and obstructive sleep apnea. As a reflection of the rising global incidence of obesity, there has been a corresponding increase in the number of obese patients undergoing surgery in general and bariatric surgery in particular.

Body mass index is calculated by the division of the subject’s weight in kilograms (kg) by the height in meters squared (m^2^). A person with a BMI of 20–25 kg/m^2^ is normal, whereas a BMI of 26–29.9 kg/m^2^ is called overweight. A patient with a BMI of 30–39.9 kg/m^2^ is defined as obese and is counted as extreme/morbid obese with a BMI > 40 kg/m^2^. An individual with a BMI > 50 kg/m^2^ is superobese, and if with a BMI > 60 kg/m^2^ is supersuperobese. The risk of developing obesity-related conditions is based on BMI, the higher the BMI, the higher the risk (Soleimanpour et al. 2017).

Obesity is an important determinant of respiratory mechanics in patients under general anesthesia by decreased compliance, decreased functional residual capacity, and increased respiratory system resistance. Changes in lung function after insufflations pneumoperitoneum and positioning of the patient remained approximately constant throughout surgery (Rauh et al. 2001).

Pneumoperitoneum and patient BMI had the most important effect on respiratory mechanics. BMI is the main risk factor for decreased lung compliance after anesthesia induction and pneumoperitoneum (Tomescu et al. 2017).

Bariatric surgery is thought to be the only effective long-term treatment for patients with BMI ≥ 40 or ≥ 35 with comorbidities. Besides the significant weight loss, bariatric surgery offers the patient additional advantages. Recent studies have shown that it can improve type 2 diabetes, hypertension, and dyslipidemia. Bariatric surgery is increasing day by day, especially because of the inadequacy of non-surgical methods of weight loss (exercise, diet, and lifestyle modification) (Dixon et al. 2008).

As the risk of anesthesia and surgery is greater in morbidly obese patients than in the normal weight population, anesthesiologists should be more familiar with the clinical management of obese patients for all surgery types, especially for weight reduction procedures (Dixon et al. 2008).

The present study compared the inverse ratio volume-controlled ventilation (I:E ratio 2:1) to the conventional volume-controlled ventilation with an I:E ratio of 1:2 in morbidly obese patients undergoing laparoscopic sleeve gastrectomy operations. The study reveals an increase in arterial PaO_2_, mean airway pressure, and compliance in the inverse ratio group together with a significant decrease in peak airway pressure, and plateau pressure, with no statistically significant change in hemodynamics either mean arterial pressure or heart rate.

Similar results were observed in another study done by Zhang and Zhu; they compared the IRV to the VCV in morbidly obese patients undergoing laparoscopic gynecological surgery in a Trendelenburg position 30° and showed that there was an increase in PaO_2_ and mean airway pressure and a decrease in peak and plateau pressures, with no statistically significant changes in hemodynamics (Zhang and Zhu, 2016).

Mousa 2013 also pointed to similar results in his crossover study equal ratio ventilation (1:1) improves arterial oxygenation during laparoscopic bariatric surgery; there was a decrease in peak airway pressure together with a significant increase in compliance and mean airway pressure. But that study was assessing the prolongation in I:E ratio in pressure control mode of ventilation not volume control (Mousa 2013).

According to the metanalysis done by Souza et al. that evaluated different ventilator strategies for morbidly obese patients undergoing bariatric surgery, they stated that an I:E ratio of 1:1 achieved greater lung compliance than an I:E ratio of 1:2 (Souza et al. 2020).

Tweed and Lee studied the effect of IRV during orthopedic surgery under general anesthesia and revealed that there is no beneficial effect for increasing Pmean by time-cycled IRV regarding gas exchange, and this is against our study results (Tweed and Lee 1991).

Prolongation of the inspiratory time together with slowing the inspiratory flow contribute to the decrease in the peak airway pressure in IRV. It is well known that high peak airway pressure leads to barotrauma and lung injury through the generation of elevated alveolar shear forces (Zhang and Zhu, 2016).

This prolongation of inspiratory time is beneficial only when a significant amount of recruitable lung units is found. This might be why previous studies failed to show or showed minimal effects of IRV in normal-weight patients under general anesthesia (Kim et al. 2013).

During pneumoperitoneum, an increase in the intrathoracic pressure occurs resulting in a decrease in lung compliance; this leads to diminution of lung volumes and thus increase of Ppeak. This consequently causes atelectasis in the dependent parts of the lungs (Nguyen and Wolfe 2005).

Inverse ratio ventilation is a well-known technique used in ARDS for improving arterial oxygenation. It increases the Pmean, reduces arteriovenous shunting, recruits atelectatic alveoli, and decreases dead space ventilation. Prolongation of the I:E ratio allows more time to reach the targeted TV with a consequent decrease in Ppeak (Mousa 2013).

IRV might lead to air trapping in the lungs with the formation of what is called intrinsic PEEP or auto-PEEP. Also, mechanical ventilation with conventional I:E ratio generated auto-PEEP possibly because of hyperinflation and high airway pressure. In addition, PEEP can improve oxygenation by increasing Pmean. So IRV would improve oxygenation. IRV may lead to auto-PEEP and that is thought to have advantageous effects on pulmonary mechanics and improve oxygenation (Zhang and Zhu, 2016).

The hemodynamics changes due to positioning have been extensively studied. Falabella et al.’s study did not find an increase in heart rate but found an increase in the mean arterial pressure and systemic vascular resistance in steep Trendelenburg position with pneumoperitoneum. This study also stated that there is a decrease in the cardiac output in the reverse Trendelenburg position secondary to a decrease of the venous return (Falabella et al. 2007)

Zhang and Zhu who compared the effect of inverse ratio ventilation and conventional volume-controlled ventilation on the cardiopulmonary functions and on the cytokine concentration bronchoalveolar lavage found that there were no significant hemodynamic changes in the two groups (Zhang and Zhu, 2016).

The generation of auto-PEEP might impede the venous return leading to the affection of hemodynamics, but our results showed that there is no statistically significant hemodynamic instability, and this goes in accordance with a study done by Sinha et al. that revealed that the pressure-controlled IRV (I:E 1.5:1) did not affect hemodynamics (Sinha et al. 2012).

To our knowledge, this study is the first to assess the inverse ratio volume control ventilation in bariatric surgery with reverse Trendelenburg position; all previous studies either used the pressure-controlled mode or just prolonged the I:E ratio; according to the metanalysis done by Souza et al., there is no advantage of PCV above VCV in morbidly obese patient ventilation.

The limitations of our study is that detection of atelectasis was not included as oxygenation alone is not an indicator of atelectasis in pneumoperitoneum

## Conclusion

In conclusion, this study found out that IRV of volume control mode is superior to conventional ratio VCV in morbidly obese patients undergoing laparoscopic sleeve gastrectomy as it increases the lung compliance, partial pressure of oxygen, and mean airway pressure together with a decrease in peak and plateau pressures.

## Data Availability

The datasets used and/or analyzed during the current study are available from the corresponding author on reasonable request.

## Abbreviations

ASA: American society of anesthesiologists classification
BMI: Body mass index
CD: Dynamic compliance
CO_2_: Carbon dioxide
CS: Static compliance
FiO_2_: Fraction inspired oxygen
GERD: Gastroesophageal reflux disease
HR: Heart rate
I:E: Inspiratory to expiratory
IRV: Inverse ratio ventilation
MAP: Mean arterial blood pressure
OR: Operation room
OSA: Obstructive sleep apnea
PaO_2_: Arterial oxygen tension
PEEP: Positive-end expiratory pressure
Pmean: Mean pressure
PO_2_: Partial pressure of oxygen
Ppeak: Peak pressure
Pplat: Plateau pressure
*P*-value: Probability
RCT: Randomized controlled trial
SD: Standard deviation
SPSS: Statistical package for social science
T0: Preoperative
T1: Immediately after intubation
T2: Before pneumoperitoneum
T3: After pneumoperitoneum
VCV: Volume-controlled ventilation
Vt: Tidal volume
